# Anti-thrombosis Effects and Mechanisms by Xueshuantong Capsule Under Different Flow Conditions

**DOI:** 10.3389/fphar.2019.00035

**Published:** 2019-02-07

**Authors:** Shuxian Han, Ying Chen, Jinyu Wang, Qian Zhang, Bing Han, Yimeng Ge, Yanhua Xiang, Rixin Liang, Xiaoxin Zhu, Yun You, Fulong Liao

**Affiliations:** ^1^Institute of Chinese Materia Medica, China Academy of Chinese Medical Sciences, Beijing, China; ^2^Harbin Zhenbao Pharmaceutical Co., Ltd., Harbin, China

**Keywords:** shear stress, endothelial cells, thrombosis, inflammation, Xueshuantong capsule

## Abstract

Xueshuantong capsule (XST) is a patented traditional Chinese medicine used for the prevention and treatment of thrombosis. The molecular mechanism of anti-thrombotic effect of XST was investigated through the cross-talk among the platelets/leukocytes, endothelial cells (ECs), and flow shear stress. The Bioflux 1000 system was used to generate two levels of shear stress conditions: 0.1 and 0.9 Pa. Bioflux Metamorph microscopic imaging system was used to analyze the adhesion cell numbers. Protein expressions were detected by western blotting and flow cytometry. The flow-cytometry results showed that under 0.1 Pa flow, XST decreased ADP induced platelets CD62p surface expression in a concentration-dependent manner. Under 0.9 Pa flow, XST at a concentration of 0.15 g⋅L^-1^ reduced the platelets activation by 29.5%, and aspirin (ASA) showed no inhibitory effects. XST showed similar efficiency on monocytes adhesion both under 0.1 and 0.9 Pa flow conditions, and the inhibition rate was 30.2 and 28.3%, respectively. Under 0.9 Pa flow, the anti-adhesive effects of XST might be associated with the suppression of VE-cadherin and Cx43 in HUVECs. Blood flow not only acts as a drug transporter, but also exerts its effects to influence the pharmacodynamics of XST. Effects of XST on inhibiting platelets activation and suppressing platelets/leukocytes adhesion to injured ECs are not only concentration-dependent, but also shear stress-dependent. The mechanic forces combined with traditional Chinese medicine may be used as a precise treatment for cardiovascular diseases.

## Introduction

The concept of Virchow’s triad regarding the pathogenesis of thrombosis (perturbations in blood, blood vessel, and/or blood flow) has provided an outline for understanding thrombotic diseases 150 years ago (Wolberg et al., 2012). Thrombotic events occurring in either arteries or veins are considered as the primary causes of fatal perioperative cardiovascular events. Blood composition is the well studied component of the triad (Wolberg et al., 2012). Platelet has been increasingly recognized as a multipurpose cell, and has highly specialized adhesive mechanisms that enable cell-matrix and cell-cell interactions throughout the thrombus formation. Activated platelets express and release molecules such as α-granule and muramidase, leading to the activation and secretion of leukocytes (Gawaz et al., 1998). Leukocytes participate in thrombosis by generating procoagulant material and contributing to the formation of arterial thrombi overlying the site of vessel rupture (Laura and Patricia, 2016). Vessel wall components, especially endothelial cells (ECs), contribute at least two essential functions during the process of coagulation. Firstly, cellular adhesion molecules including VCAM-1, VE-cadherin, or Cx43 (Liu et al., 2015) are exposed during vascular injury, and recruit leukocytes and platelets to the sites of vascular damage. Secondly, the vessel wall components provide coagulation complexes with a surface upon for assembling. The effects of blood flow on clotting have drawn the attention of many researchers. Blood flow in the vascular system is typically characterized by shear stress, which is defined as the force per unit area between adjacent layers of fluid. The structure and function of ECs could be influenced by shear stress, which might in turn modulate the expressions of genes and proteins associated with thrombotic diseases (Chatzizisis et al., 2007; Chiu et al., 2009; Chiu and Chien, 2011). In the venous system, altered shear stress may induce the dysfunction of ECs, leading to the development of several chronic venous diseases, such as varicose veins and deep venous thrombosis (Bergan et al., 2006; Chiu and Chien, 2011).

Panax notoginseng (PNS), a famous ancient traditional Chinese herb, has been reported with various beneficial effects, such as promotion of blood circulation, removal of the blood stasis, relieve pain, and hemostasis. It is widely used in clinical earliest records as found in the Ming dynasty and described its function medically in the Compendium of Materia Medica (Ben Cao Gang Mu). Total saponins are considered to be the most important and active ingredient of PNS, and contains a variety of monomer saponins including ginsenoside Rg_1_, Rb_1_, Re, Rd, Rb_2_, Rb_3_, Rc, and notoginsenoside R_1_, R_2_, R_3_, R_6_ (Wan et al., 2006; Shi et al., 2012). In recent years, many pharmacological effects of PNS were found, such as platelet aggregation inhibition, anti-myocardial ischemia, anti-cerebral ischemia, anti-inflammation, anti-oxidation, protecting ECs (Lau et al., 2009; Chen et al., 2011; Jia et al., 2014). Also PNS inhibited the oxidized low-density lipoproteins (ox-LDL) induced platelets adhered to ECs (Wang M.M. et al., 2016). Xueshuantong capsule (XST) is an approved traditional Chinese medicine used for the treatment of stroke, hemiplegia and heartache caused by thrombosis or blood stasis, and each capsule of it contains 100 mg PNS. Since thrombus formation is a complex, multifactorial and interactive process, many studies aimed to capture the interplay among the compounds (herbs), blood cells, and fluid environment. Xu et al. (2010) developed a computational model that invoked the cellular clotting model to represent platelets in flow. You et al. (2014) designed the inflammation model of leukocytes adhesion and transmigration to ECs under controlled shear stress *in vitro*.

Our previous study has focused on the molecular mechanism of XST anti-thrombotic effect through the cross-talk among the blood cells, blood vessel and blood flow by using the method of biomechanopharmacology (Liao et al., 2006). The results showed that shear stress has influenced the effects of XST anti-platelets adhesion to TNF-α injured HUVECs. Also found that XST suppressed TNF-α induced endothelial VCAM-1 expression significantly under 0.1 Pa flow condition, but without the effects on its expression under 0.9 Pa flow condition (Han et al., 2017). Since thrombus formation is a very complex process that involve activation of platelets, leukocytes (Gawaz et al., 1998; Laura and Patricia, 2016), ECs and blood flow, the present study tried to explore the effects of XST from the comprehensive scene to find the specific molecular pathways involved in thrombosis prevention and therapy under specific flow shear stress or static conditions. Understanding the mechanism of XST will allow us to design the target, and therefore a safer and more effective therapeutics to treat thrombosis can be produced. The aim of this study is to understand how the efficacy and mechanisms of XST anti-thrombus under different flow conditions. The hypothesis was tested using a factorial design of two factors: XST and shear stress, and two levels of each factor. Our investigation strategy was as follows: first, to evaluate the effects of XST on platelets activation induced by adenosine diphosphate (ADP) under flow; second, to investigate the effects of XST on monocytic THP-1 cell adhesion in TNF-α injured EC monolayers under flow; third, to find the underlying molecular mechanisms of XST on the expression of ECs of VE-cadherin and Cx43 under flow and static conditions.

## Materials and Methods

### Materials

Xueshuantong capsule were obtained from Harbin Zhenbao Pharmaceutical Co., Ltd. (China, Lot no.20140133), Aspirin (ASA) was purchased from Sigma. Human umbilical vein endothelial cell (HUVEC), endothelial cell medium (ECM), endothelial cell growth supplement (ECGS), fetal bovine serum (FBS) and antibiotics were purchased from Sciencell (CA, United States). Dimethyl sulfoxide (DMSO) and methyl thiazolyl tetrazolium (MTT) were purchased from Sigma (United States). ADP was purchased from Techlink Biomedical (Beijing, China). Anti-Cx43 antibody was purchased from Santa Cruz Biotechnology, Inc. (CA, United States). Anti-VE-cadherin antibody was purchased from Abcam (Boston, MA, United States). TNF-α was purchased from PeproTech APAC (Rehovot Israel). FBS and Trypsin EDTA (0.25%) were purchased from Gibco (CA, United States). RPMI 1640 medium, penicillin, and streptomycin were purchased from Hyclone (UT, United States). Bioflux 48-well plates (24 flow channels) were obtained from Fluxion Biosciences (CA, United States).

### Preparation of Test Drugs

The powder of XST was dissolved in 0.9% saline water. After 30 min of ultrasonic dissolution, the solution was centrifuged at 3500 r⋅min^-1^ for 5 min. Then, the supernatant was filtrated by 0.2 μm filter membrane, and the stock concentration was 66.67 g⋅L^-1^. ASA powder was dissolved in PBS to a stock concentration of 50 mmol⋅L^-1^, with the pH value adjusted to 7.2–7.4.

The HPLC method was used to quantify five representative constituents of XST solution, including PNS saponin R_1_, Rg_1_, Re, Rb_1_, and Rd. The methods and sample preparation were performed according to the National Drug Standards of China Food and Drug Administration (NO.WS-10696(ZD-0696)-2002-20127).

An Agilent 1200 HPLC system (Agilent, PA, United States) was employed to analyze the constituents of XST. The separation was conducted on a Kromasil C18 column (250 mm × 4.6 mm i.d., 5 μm, Kromasil, SE) at a flow rate of 1.5 mL⋅min^-1^. A gradient elution system consisting of acetonitrile (A) and water (B) was performed using the following gradient program: 0–20 min, 20% A; 20–45 min, 20–46% A; 40–55 min, 46–55% A; 55–60 min, 55% A. Column temperature and the detection wavelength were set 25°C and 203 nm, respectively. HPLC fingerprint of the XST capsule was shown in Supplementary Figure S1.

The content of the five compounds in XST stock solution (66.67 g⋅L^-1^) was calculated by external standard method, and the concentrations of notoginsenoside R_1_, ginsenoside Rg_1_, ginsenoside Re, ginsenoside Rb, and ginsenoside Rd_1_ were 8.448, 22.464, 3.024, 25.404, and 5.412 g⋅L^-1^ respectively.

In the experimental system, in XST solution at concentrations of 0.60 g⋅L^-1^, the concentration of five compounds notoginsenoside R_1_, ginsenoside Rg_1_, ginsenoside Re, ginsenoside Rb, and ginsenoside Rd_1_ were 0.076, 0.202, 0.027, 0.229, and 0.049 g⋅L^-1^ respectively.

### Cell Culture and Platelets Preparation

The primary HUVECs were obtained from Sciencell (CA, United States, Lot NO.15536). HUVECs were cultured in ECM containing 5% FBS, 1% ECGs, and 1% antibiotics, and the third to eighth passages were used for all the experiments. The human monocytic leukemia cell line THP-1 (ATCC; Manassas VA, United States) was maintained in 1640 medium supplemented with 10% FBS and 1% antibiotics. The study was approved by the Independent Institute Research Ethics Committee at the Beijing Hospital and conducted in accordance with the Declaration of Helsinki. The platelets were obtained from the consenting healthy adult volunteers. Fresh blood was mixed with anticoagulant acid citrate dextrose (ACD-A) at a volume ratio of 9:1. Platelet-rich plasma (PRP) was obtained following blood sample centrifugation at 200 × *g* for 10 min. PRP samples with 5 mmol⋅L^-1^ ACD (2.5% trisodium citrate, 2.0% glucose, 1.5% citric acid) and 5 mmol⋅L^-1^ EDTA were again centrifuged at 400 × *g* for 10 min. Platelets were washed with Tyrode buffer (138 mmol⋅L^-1^ NaCl, 3.3 mmol⋅L^-1^ NaH_2_PO_4_, 2.9 mmol⋅L^-1^ KCl, 1 mmol⋅L^-1^ MgCl_2_, 5.5 mmol⋅L^-1^ glucose, 20 mmol⋅L^-1^ HEPES, pH 7.2), and were centrifuged at 400 × *g* for 10 min. The platelets concentration was adjusted as needed with Tyrode buffer. All platelet preparations were conducted at room temperature.

### Cytotoxic Assay

Cell viability assays were conducted using MTT assay. Briefly, log phase HUVECs were plated in 96-well plates separately at a density of 1 × 10^4^ cells per well and incubated for 24 h. Then, the vehicle and five concentrations of XST (0.12, 0.30, 0.60, 0.75, 1.88, 3.00 g⋅L^-1^) were co-incubated with the cells for 24 h. Five duplicate wells were set up in each sample. Two independent experiments were carried out. After treatment, the cells were incubated with MTT at a final concentration 0.50 g⋅L^-1^ for 4 h at 37°C. The media was carefully removed from each well and then 150 μL of DMSO was added. The plates were gently agitated and the OD was determined using a microplate reader at 490 nm (M5, Molecular Devices, CA, United States).

### Flow Cytometric Analysis

To assess the effects of XST on platelets activation, the washed platelets were pretreated with varied concentrations of XST (0.15, 0.30, and 0.60 g⋅L^-1^) or ASA (1 mmol⋅L^-1^) under 0.1 or 0.9 Pa flow condition at 37°C for 5 min. The control and model groups were treated with same amount of vehicle. ADP (10 μmol⋅L^-1^) was used to activate the platelets at room temperature for 5 min, and the control group was treated with the same volume of vehicle. Then the platelets were incubated with antibodies against CD61 (FITI-conjugated) and CD62p (PE-conjugated) at room temperature for 30 min. Platelets were again centrifuged at 400 × *g* for 8 min and resuspended in Tyrode buffer. After that, flow cytometric analysis was performed to detect the activation of platelets, and approximately 1 × 10^6^ platelets were evaluated per sample.

### THP-1 Adhesion Assay Under Shear Stress

Human umbilical vein endothelial cells were cultured under flow conditions using the Bioflux 1000 flow system (Fluxion Biosciences, CA, United States) (Han et al., 2017). In brief, the channels of Bioflux 48-well plates were coated with 60 mg⋅L^-1^ rat tail collagen. HUVECs were seeded and incubated in the plates overnight, and were treated with TNF-α (20 μg⋅L^-1^; incubation time: 2 h, at 37°C) in the absence or presence (12-h preincubation) of XST (0.30 g⋅L^-1^) or ASA (1 mmol⋅L^-1^). THP-1 cells at a density of 5 × 10^9^ cells⋅L^-1^ were introduced into the viewing channels at 0.2 Pa shear stress. Then, the THP-1 cell suspension was continued flowing under controlled flow conditions at 0.02 Pa shear stress for 60 min, and images were captured by time-lapse microscopy (exposure time 20 ms). THP-1 cell adhesion numbers were measured by Bioflux Montage software. HUVECs were washed with ice-cold PBS, and cleaved in RIPA buffer containing proteinase inhibitors (1 mmol⋅L^-1^ PMSF). The lysis solution was used for western blot analysis. Three independent experiments were carried out same as described above.

### Platelets Adhesion Assay Under Shear Stress

As previously described (Han et al., 2017), HUVECs were seeded on the viewing channels and incubated overnight, and pretreated with XST (0.30 g⋅L^-1^) or ASA (1 mmol⋅L^-1^), TNF-α (20 μg⋅L^-1^). Platelets at 2.5 × 10^11^ cells⋅L^-1^ were introduced into the viewing channels at 0.2 Pa shear stress. Then the platelets suspension was continued flowing under controlled flow conditions at 0.01 Pa for 45 min, and images were captured by time-lapse microscopy (exposure time 150 ms). Platelets adhesion numbers were measured by Bioflux Montage software. HUVECs were cleaved by lysis buffer and the lysis solution was used for western blot analysis.

### Western Blot Assay

Human umbilical vein endothelial cells were incubated with TNF-α (20 μg⋅L^-1^; incubation time: 2 h, at 37°C) in the absence or presence (12-h preincubation) of XST (0.30 and 0.60 g⋅L^-1^) or ASA (1 mmol⋅L^-1^). After washing three times with cold PBS, cell lysis (RIPA buffer containing 1 mmol⋅L^-1^ PMSF) was performed. After centrifugation for 5 min at 12000 r⋅min^-1^ at 4°C, the protein concentration in the supernatant was determined with a BCA assay kit according to the manufacturer’s instructions.

Equivalent amounts of proteins (5–15 μg/lane) were separated by 8% sodium dodecyl sulfate polyacrylamide gel electrophoresis (SDS-PAGE) and subsequently transferred to PVDF membranes. Each membrane was blocked with 5% skim milk in Tris Buffered Saline with Tween-20 (TBST) saline (20 mmol⋅L^-1^ Tris-HCl, pH 7.5, 137 mmol⋅L^-1^ NaCl, and 0.1% Tween 20) at room temperature for 2 h and then incubated with indicated primary antibodies: rabbit monoclonal anti-VCAM-1 (1:10000, Santa Cruz Biotechnology, Inc., CA, United States), rabbit monoclonal anti-VE-Cadherin (1:10000, Abcam, MA, United States), rabbit monoclonal anti-Cx43 (1:5000, Santa Cruz Biotechnology, Inc., CA, United States), and mouse monoclonal anti-β-actin (1:10000, Santa Cruz Biotechnology, Inc., CA, United States) overnight at 4°C. After being washed with TBST, membranes were incubated with horseradish peroxidase conjugated secondary antibody for 2 h at room temperature. Bands were visualized by enhanced chemiluminescence (ECL) (Millipore) and analyzed by an ECL detection system (Syngene, Cambridge, United Kingdom). The mean density of each band was analyzed using Image J. Each experiment was carried out for three times.

### Statistical Analysis

All the data was represented as means ± SD of at least three independent experiments performed in at least triplicate. Statistical analysis was carried out using the Statistical Package for the Social Sciences (SPSS, Inc., Chicago, IL, United States). Statistical significance of the groups was determined by analysis of variance (ANOVA) followed by LSD test for experiments carried out in static condition and for HUVECS viability. The GLM univariate procedure was performed for a two-way ANOVA to test for significance across flows, and a LSD *post hoc* test was used to show the differences for each pair of factor levels. A *P*-value <0.05 was considered significant.

## Results

### Effects of XST on the Viability of HUVECs

**Figure 1:**
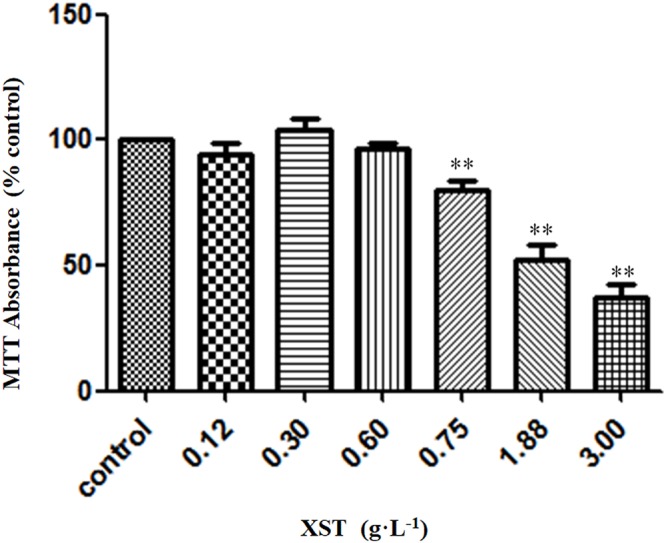
Effects of XST on viability of HUVECs. Cells were treated with 0.9% saline water or XST for 24 h. Cell growth was measured by MTT assay. The data was presented as means ± SD, *n* = 5. ^∗∗^*P* < 0.01 vs. control.

The effects of XST at different concentrations on HUVECs viability were examined by MTT assay. XST of up to 0.60 g⋅L^-1^ showed no significant effect on the growth of either cells (Figure 1). The safe dose range of XST was determined. In the pilot experiments, the dosage of XST (0.3 and 0.6 g⋅L^-1^) was used for the following experiments.

### Effects of XST on Platelets Activation

**Figure 2:**
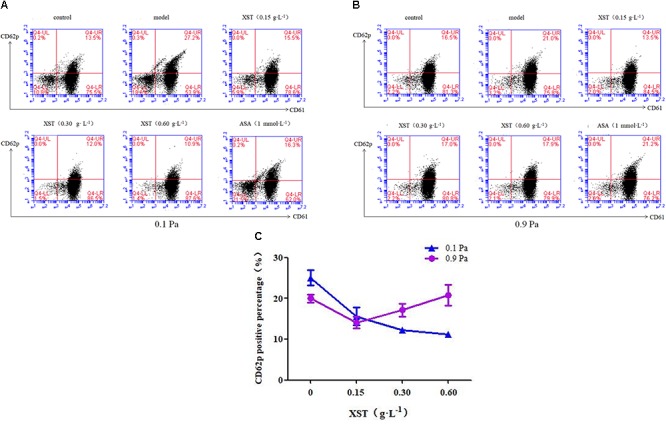
Comparisons of platelets activation between groups. Representative scatter diagrams showing platelet CD62p expression **(A)** under 0.1 Pa flow condition, and **(B)** under 0.9 Pa flow condition. **(C)** Comparison of CD62p positive percentage between groups under different flow conditions. The percentages of CD61^+^ CD62p^+^ platelets are depicted in the upper right quadrants of the contour plots, the CD61^+^ CD62p^-^ and the CD61^-^ CD62p^+^ platelets are depicted in the lower right and upper left quadrants, respectively, and CD61^-^ CD62p^-^ platelets are depicted in the lower left quadrants. The data was presented as means ± SD, *n* = 3; data was analyzed by two-way ANOVA as described in Methods; analysis of simple effects.

To determine how XST influences platelets activation under flow conditions, we measured CD62p (P-selectin) exposure, a marker of α-granule release, on the platelet surface by flow cytometry (Figure 2). Under 0.1 Pa flow condition, 13.57 ± 0.60% of platelets in the control group expressed CD62p on the membrane surface, while 5-min stimulation with ADP (10 μmol⋅L^-1^) increased the number of CD62p-positive platelets by up to 25.07 ± 1.91% (Figure 2A, model vs. control). However, under 0.9 Pa flow condition, ADP stimulation increased the number of CD62p-positive platelets by up to 20.00 ± 0.95%, while in the control group 15.40 ± 2.63% of platelets expressed CD62p on the membrane (Figure 2B). ADP stimulation raised CD62p expression by 84.77 ± 14.11% under 0.1 Pa, and by 29.87 ± 6.19% under 0.9 Pa.

In this study, two levels of shear stress were set, one is pathological lower shear stress of 0.1 Pa and the other is physiological artery shear stress of 0.9 Pa. A positive interactive effect of XST and shear stress on platelets CD62p exposure was confirmed by two-way ANOVA (*F* = 24.567, *P* < 0.001). Main effects for XST and shear stress were also significant [(*F* = 31.492, *P* < 0.001), (*F* = 9.092, *P* = 0.008) respectively]. XST inhibited the activation of platelets in a dose-dependent manner under 0.1 Pa flow condition, and the inhibitory rate was 37.8, 51.1, and 55.6% respectively, with concentrations of 0.15, 0.30, and 0.60 g⋅L^-1^. Under 0.9 Pa flow condition, XST of 0.15 g⋅L^-1^ reduced the platelets activation significantly by 29.5%; 0.30 and 0.60 g⋅L^-1^ of XST showed no significant reduction. The switch from 0.1 to 0.9 Pa resulted in the less efficacy of XST. In addition, ASA reduced CD62p expression by 36.2% under 0.1 Pa flow condition.

### Effects of XST on HUVECs and Platelets Adhesion

**Figure 3:**
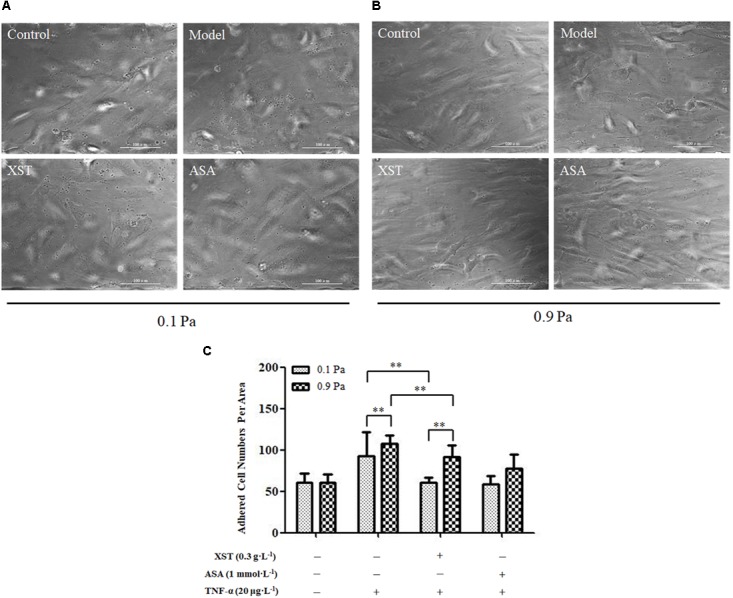
Comparisons of platelets adhesion between groups. **(A)** under 0.1 Pa flow condition, and **(B)** under 0.9 Pa flow condition (magnification × 200). **(C)** Histogram demonstrating the platelets adherence on activated HUVECs under different flow conditions. The data was presented as means ± SD, *n* = 9; data was analyzed by two-way ANOVA as described in Methods; analysis of simple effects. ^∗∗^*P* < 0.001.

Two-way ANOVA revealed no significant interaction between XST and shear stress on platelets adhesion (*F* = 1.746, *P* = 0.196) but very significant main effects for XST (*F* = 16.798, *P* < 0.001) and shear stress (*F* = 15.566, *P* < 0.001), indicating that these factors acted independently. XST pretreatment demonstrated an obvious reduction in adhesion platelets (60.78 ± 6.28) compared with those in TNF-α activated HUVECs (92.23 ± 29.20), and the average inhibitory rate was 34.1% under 0.1 Pa flow condition. Compared with the model group, platelets adhesion numbers in XST treatment group were decreased significantly (107.44 ± 10.01 vs. 91.33 ± 14.81), and the average inhibitory rate was 15.0% under 0.9 Pa flow condition (Han et al., 2017). The levels of shear stress were also associated with the platelets adhesion. In this article, we showed the images and histogram of platelets adhesion under different flow conditions (Figure 3).

### Effects of XST on HUVECs and THP-1 Adhesion

**Figure 4:**
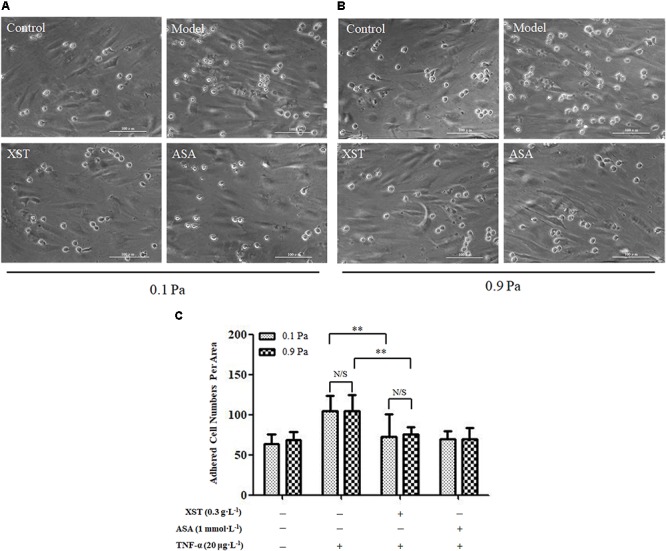
Comparisons of THP-1 adhesion between groups. **(A)** under 0.1 Pa flow condition, and **(B)** under 0.9 Pa flow condition (magnification × 200). **(C)** Histogram demonstrating the THP-1 cells adherence on activated HUVECs under different flow conditions. The data was presented as means ± SD, *n* = 9; data was analyzed by two-way ANOVA as described in Methods; analysis of simple effects. ^∗∗^*P* < 0.001.

There was no obvious interactive effect between XST and shear stress on THP-1 adhesion by two-way ANOVA (*F* = 0.024, *P* = 0.877). The shear stress main effect was not significant (*F* = 0.024, *P* = 0.877) indicating that THP-1 adhesion numbers were similar under 0.1 and 0.9 Pa shear stress (104.89 ± 20.35 vs. 104.89 ± 19.53, respectively). Main effect for XST was significant (*F* = 20.593, *P* < 0.001) indicating that XST inhibited THP-1 cells adhesion on TNF-α injured endothelium markedly. Under 0.1 Pa flow condition, XST pre-treatment demonstrated an obvious reduction in adhesion platelets (73.11 ± 27.49) compared with those in TNF-α activated HUVECs (104.89 ± 20.35). Under 0.9 Pa flow condition, XST pre-treatment also reduced THP-1 adhesion (75.22 ± 9.92) compared with the model group (104.89 ± 19.53), and the average inhibitory rate was 28.3%. Under two levels of shear stress, ASA reduced THP-1 adhesion by a rate of 33.2 and 33.9%, respectively (Figure 4).

### Effects of XST on VE-Cadherin, Cx43 Expression in TNF-α-Treated HUVECs Under Static Condition

**Figure 5:**
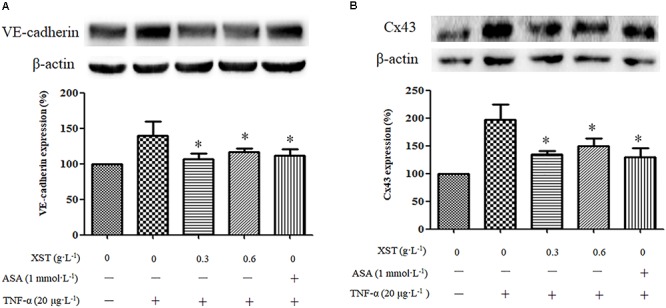
Comparisons of VE-cadherin and Cx43 expression between groups under static condition. **(A)** The expression and density of VE-cadherin under static condition were analyzed by western blotting. **(B)** The expression and density of Cx43 under static condition were analyzed. The data was presented as means ± SD, *n* = 3. ^∗^*P* < 0.05 vs. model.

The western blot results demonstrated that the XST (0.3, 0.6 g⋅L^-1^) treatment decreased the expression of VE-cadherin and Cx43 significantly (Figure 5). XST at concentrations of 0.30 and 0.60 g⋅L^-1^ showed inhibitory effects on the expression of these membrane proteins. Treatment with 0.30 g⋅L^-1^ XST decreased VE-cadherin and Cx43 expression by 23.5 and 31.9%, and 0.60 g⋅L^-1^ XST reduced their expression by 16.6 and 24.0%, respectively (Figure 5B).

### Effects of XST on VE-Cadherin and Cx43 Expression in TNF-α-Treated HUVECs Under 0.1 and 0.9 Pa Flow Conditions

Western blotting was performed to elucidate the potential underlying mechanisms of the inhibitory effects of XST on THP-1 adhesion under different flow conditions.

**Figure 6:**
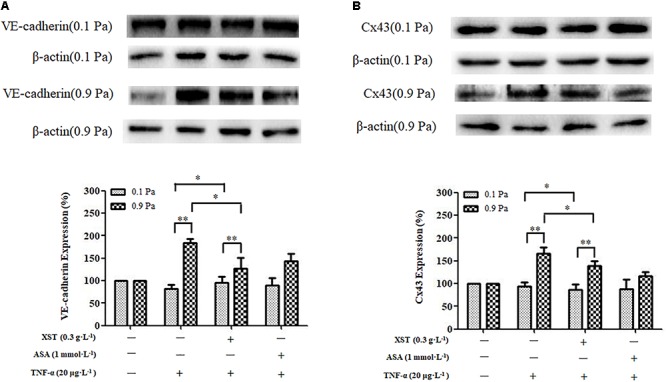
Comparisons of VE-cadherin and Cx43 expression between groups under 0.1 and 0.9 Pa flow conditions. **(A)** The expression and density of VE-cadherin under 0.1 and 0.9 Pa flow conditions were analyzed by western blotting. **(B)** The expression and density of Cx43 under 0.1 and 0.9 Pa flow conditions were analyzed. The data was presented as means ± SD, *n* = 3; data was analyzed by two-way ANOVA as described in Methods; analysis of simple effects. ^∗∗^*P* < 0.001; ^∗^*P* < 0.05.

A positive interactive effect between XST and shear stress on ECs VE-cadherin expression (*F* = 30.676, *P* < 0.001) was confirmed by two-way ANOVA. Main effects for XST and shear stress were also significant. Shear stress affected the VE-cadherin expression in HUVECs remarkably (*F* = 115.519, *P* < 0.001). Under 0.9 Pa flow condition, treatment with 0.3 g⋅L^-1^ XST decreased the endothelial expression of the VE-cadherin remarkably, and the inhibitory rate was 30.6%; the effect of XST on VE-cadherin expression was not obvious under 0.1 Pa flow condition. The significant interactive effects between XST and shear stress indicated that XST inhibited VE-cadherin expression was influenced by shear stress, that is, the XST exhibited stronger inhibitory effect on VE-cadherin expression under 0.9 Pa flow condition (Figure 6).

Two-way ANOVA revealed no obvious interaction between XST and shear stress on ECs Cx43 expression but very significant main effects for XST (*F* = 6.819, *P* = 0.031) and for shear stress (*F* = 96.269, *P* < 0.001), indicating that these factors acted independently. Under 0.9 Pa flow condition, treatment with 0.3 g⋅L^-1^ XST significantly decreased the expression of the Cx43, and the inhibitory rate was 16.0%; and the effects of XST Cx43 expression were not obvious under 0.1 Pa flow condition. The switch from 0.1 to 0.9 Pa shear stress resulted in a significantly increased ECs Cx43 expression (Figure 6).

ASA did not show visibly inhibitory effects on VE-cadherin and Cx43 expression either (Figure 6).

## Discussion

The present study investigated the effects and possible mechanisms of XST capsule on platelets activation, leukocytes adhesion, and endothelial protein expression under different flow conditions. Virchow’s triad (blood flow, vessel wall, blood constitutes) has been considered as the framework of biomechanopharmacological paradigm and interactions of shear stress, endothelium, and platelets/leukocytes that play important roles in thrombotic disease (Liao et al., 2006; Wolberg et al., 2012). Endothelium mediates pathological consequences and protective responses in both acute and chronic inflammation, and cardiovascular disorders (Han et al., 2017). Platelets and leukocytes adhesion to the activated ECs lining of the blood vessels is the first step to perform the tasks related to injury and inflammation. It has been widely proposed that the anti-adhesive therapy is a rational and effective approach for the treatment of thrombotic diseases (Barreiro et al., 2010). Many physiological or pathological processes occur under flow conditions, atherosclerosis plaque forming on vasculature region with low shear rate, cancer cells circulating throughout the body (Gakhar et al., 2013), and turbulence flow activates platelet biogenesis in mice (Ito et al., 2018). It is established that physiological flow has a profound impact on many biological process, yet much research is still conducted *in vitro* without the presence of flow. BioFlux 1000 system introduce flow to research (Conant et al., 2011; Wang S.P. et al., 2016) and generate two levels of shear stress at the same time, emulating *in vivo* flow conditions and to reveal drug efficacy under controlled flow shear stress (Supplementary Figure S2). The present experiments were carried out under two levels of shear stress in parallel to study the effects of XST with different concentrations. Some studies have shown that in humans the magnitude of shear stress ranges from 0.1 to 0.6 Pa in the venous system and from 1 to 7 Pa in arteries (Chiu and Chien, 2011). Some studies represent that blood vessels adapt their caliber via endothelium-mediated regulation to maintain a mean wall shear stress of approximately 0.5 to 2 Pa in much of the arterial system (Wolberg et al., 2012; Shobhit and Sriram, 2015). Based on these references and the present experimental condition, 0.9 and 0.1 Pa were set up as the physiological and pathological low shear stress for comparison.

XST is an approved PNS preparation which is used 600 mg orally per day in clinic. We have carried out the pre-experiment, in which the results demonstrated that 0.3 g⋅L^-1^ of XST showed effect on the inhibition of platelets adhesion, and the inhibition rate was about 25–30%. Xuesaitong Injection (Harbin Zhenbao Pharmaceutical Co., Ltd.) is also an approved PNS preparation which has the same five compounds as XST, used for the treatment of stroke, hemiplegia, and heartache caused by thrombosis or blood stasis, administrated 500 mg intravenously in clinic. It is estimated that 0.13 g⋅L^-1^ of PNS in serum after a person of 60 kg body weight was injected Xuesaitong Injection. The concentration of 0.15 g⋅L^-1^ of XST (PNS) is the link with *in vivo* concentrations in humans. Besides, according to our previous study, XST capsule of 0.6 g⋅L^-1^ showed the similar effects as Xuesaitong Injection on anti-platelets aggregation *in vitro* (Han et al., 2015). Taken together, we choose 0.15, 0.3 and 0.6 g⋅L^-1^ of XST as the experimental concentration.

Platelets are thought to be mechanical devices, and shear stress influences platelet activation, adhesion, and aggregation procedure (Kroll, 2010). CD62p is a more reliable marker for measuring shear stress induced platelets activation and it has the potential for predicting the function of platelets *in vitro* (Lu and Malinauskas, 2011). Platelets could be activated through various physiological stimuli, such as thrombin, ADP, and collagen. When platelets are activated, they adhere to the injured blood vessel walls by interaction of the glycoprotein (GP) Ib-V-IX complex with von Willebrand factor (vWF) immobilized on the exposed subendothelial matrix (Stegner and Nieswandt, 2011). We have demonstrated that XST inhibited platelet aggregation induced by ADP, collagen, and thrombin in a dose-dependent manner (Han et al., 2015). Besides, compared with ox-LDL induced platelets, PNS at 0.18 g⋅L^-1^ concentration decreased the CD62p expression significantly (Wang M.M. et al., 2016). In this study, flow cytometry was performed to quantify platelet activation with the expression of CD62p on the membrane of platelets. CD62p expression stimulated by ADP was increased by 84.77 ± 14.11% under 0.1 Pa, while by 29.87 ± 6.19% under 0.9 Pa. Two-way ANOVA analyses confirmed a significant positive interaction between XST and shear stress in reducing platelets activation, platelets CD62p exposure rates were lowered by both XST and shear stress. These demonstrated that XST and shear stress jointly affected platelet activation markedly. XST inhibited the platelets activation in a concentration-dependent manner under 0.1 Pa flow condition, and the average inhibition ratio was 37.8, 51.1 and 55.6%, respectively, with concentrations of 0.15, 0.30 and 0.60 g⋅L^-1^. Besides, ASA reduced the CD62p expression by 36.2%. Nevertheless, not all drugs, especially Chinese drugs, present a dose-dependent manner (Wang et al., 2014). Under 0.9 Pa flow condition, low dose XST of 0.15 g⋅L^-1^ reduced the platelets activation significantly by 29.5%; however, 0.30 and 0.60 g⋅L^-1^ of XST, as well as ASA showed no significant reduction. ASA did not exhibit the inhibitory effect, and was consistent with the former study where shear stress can overcome ASA inhibition of platelets activation and aggregation *in vivo* (Maalej and Folts, 1996). These results suggested that shear stress affected the inhibitory efficiency of XST on platelets activation.

To reveal the potential effects and mechanisms of XST on platelets/leukocytes adhered to TNF-α injured endothelial monolayers under flow conditions, we first established the interaction model between platelets and ECs by Bioflux 1000 system (Han et al., 2017). We have previously reported that under 0.1 Pa flow condition, XST at a concentration of 0.3 g⋅L^-1^ showed stronger inhibitory effect on the platelets adhesion than that under 0.9 Pa flow condition, and the inhibitory rate was 34.1 and 15.0%, respectively (Han et al., 2017). Two-way ANOVA showed that XST and shear stress acted on platelets adhesion independently. These data along with the evidences from the platelets CD62p expression specimens revealed that XST showed stronger anti-platelet activation and adhesion effect under low shear stress (0.1 Pa), suggesting that shear stress might influence the anti-thrombosis efficiency of XST.

Venous thrombi are formed in the setting of low flow and low shear stress (Paul and Sabine, 2005). Some pre-clinical and clinical cases reports have discussed the efficacy of XST or PNS on deep vein thrombosis (DVT). Xuesaitong Injection which has the same compounds as XST, demonstrated a potential effect on DVT in inferior vena cava thrombosis model in rabbits in a dose-dependent manner (Han et al., 2016). Our previous results showed XST significantly inhibited platelet aggregation induced by thrombin *in vitro* (Han et al., 2015). PNS orally taken at 100 mg/kg significantly reversed thrombin-induced hyper-coagulate state in rats which was accompanied by PPAR-γ protein and mRNA up-regulation in rat lung, which indicated that PNS might play a preventive role in the process of thrombosis, especially in DVT and pulmonary embolism (Shen et al., 2017). A series of case reports suggested that PNS were a feasible and effective treatment option for peripherally inserted central catheters (PICCs) related venous thrombosis in cancer patients (Huang et al., 2013). Together with the above mentioned evidence, our results revealed that XST showed higher efficacy on thrombosis inhibition under low shear stress condition than ASA did, which is consistent with the studies on the effects of ASA which is known not to have clinically significant efficacy in DVT.

Monocytes are important type of leukocytes that play an essential role in the development of arterial thrombotic diseases (Lee et al., 2014). During the early stage of thrombosis, vascular ECs were injured by pro-inflammatory factors and had low disturbed flow, leading to the recruitment of monocytes (Javier and Klaus, 2008; Burak et al., 2010). Recent results showed that XST inhibited leucocytes adhesion significantly under both 0.1 and 0.9 Pa flow conditions, and the inhibition rate was 30.2 and 28.3%, respectively. Under different flow conditions, XST showed similar efficiency on monocytes adhesion, suggesting that the main effects are exerted by XST rather than flow shear stress. In this study, we did not investigate the effects of XST on interplay between platelets and leukocytes. Direct interplay between platelets and leukocytes under flow conditions should be carried out in the near future.

To reveal the potential effects and mechanisms of XST on platelets/leukocytes adhered to TNF-α injured endothelial monolayers under flow conditions, and the adhesion related proteins VCAM-1 and Cx43, membrane vascular endothelial cadherin (VE-cadherin) expression was detected by western blotting. VCAM-1 is an important adhesion molecule that mediates monocyte recruitment to the injured endothelium (Ravi et al., 2007). Increased expression of VCAM-1 in the arterial endothelium may promote the adhesion and recruitment of inflammatory cells and platelets, contributing to the development of thrombosis (Yang et al., 2016). We have previously reported that XST suppressed the expression of VCAM-1 significantly under static and 0.1 Pa flow condition, which was superior to ASA. While under 0.9 Pa flow condition, XST showed no inhibitory effects on the expression of VCAM-1, but ASA showed (Han et al., 2017).

The adhesion molecules of vascular endothelium play key roles in thrombogenic procedure. In this study, we compared the expressions of VE-cadherin, and Cx43 of ECs under static, 0.1 and 0.9 Pa flow conditions. VE-cadherin is an endothelial-specific transmembrane component of adherent junction complexes enriched in cell membrane. Lipopolysaccharide (LPS) stimulation markedly decreased the expression of VE-cadherin in the cell membrane, but enhanced its internalization in cytoplasm, subsequently leading to the endothelial barrier disruption and vascular inflammation (Yang et al., 2015). As per our results, the expression of VE-cadherin was enhanced or internalized in TNF-α-treated ECs too. In turn, its level was attenuated by XST under static and 0.9 Pa flow condition. Two-way ANOVA revealed a significant interaction between XST and shear stress in VE-cadherin expression reduction, indicating that the VE-cadherin expression was inhibited by both XST and shear stress obviously.

Cx43 is one of the subtypes of connexins, which is a gap junction protein enriched in ECs, and it has various biological functions. Although the role of Cx43 was complex, growing evidence suggests that Cx43 is associated with inflammation in the expression of adhesion molecule and transmigration of leukocyte (Tsuchida et al., 2013; O’Donnell et al., 2014). The up-regulation of Cx43 expression enhanced monocyte-endothelium adhesion and this was markedly decreased by down-regulation of Cx43. This in turn was associated with Cx43-induced expression of VCAM-1 and ICAM-1 (Yuan et al., 2015). Our results showed that Cx43 expression was up-regulated by proinflammatory mediator TNF-α, suggesting that the function of the ECs might be disordered, followed by increasing leukocyte recruitment, while XST and ASA reduced its level under 0.9 Pa flow condition. Two-way ANOVA analysis revealed that Cx43 expression was inhibited by XST and shear stress significantly, but in this case there was no interaction suggesting independent roles for the interventions in regulating Cx43 expression.

As mentioned above, VCAM-1, VE-cadherin, and Cx43 are connected with inflammation, mediating platelets recruitment, and leukocyte adhesion on inflammatory endothelium. Our results demonstrated that XST reduced the expression of VCAM-1 (Han et al., 2017), VE-cadherin, and Cx43 under static condition, while showed different effects under different flow conditions. Under 0.1 Pa flow condition, the inhibition on platelets and THP-1 adhesion by XST were mediated through decreased VCAM-1 expression (Han et al., 2017); and under 0.9 Pa flow condition, the anti-adhesive effect of XST was associated with the suppression of VE-cadherin and Cx43. The different effects of XST on the expression of adhesion proteins under 0.1 and 0.9 Pa shear stress implied vascular biological effects of mechanic forces.

## Conclusion

In conclusion, we introduced novel modes of leukocyte-vessel wall-blood flow interaction into traditional Chinese medicine pharmacological study in thrombotic disease prevention and treatment. Our data have demonstrated that the efficacy and mechanism of XST on platelets activation and leukocytes adhesion were influenced by different shear stress conditions. Blood flow not only acts as a drug transporter, but also exerts its effects through biomechano-responses to influence the pharmacodynamics. XST inhibits platelet activation and suppresses leukocytes adhesion to injured ECs under controlled shear stress *in vitro*. Interestingly, the effects of XST are not only dose-dependent, but also shear stress-influenced. This study deepens our understanding regarding anti-thrombotic and anti-inflammatory effects of XST in clinic from the view of biomechanopharmacology.

## Ethics Statement

This study was carried out in accordance with the recommendations of Platelets Preparation Guidelines of Clinical Laboratory, Ethics Committee of the Institute of Geriatrics, Beijing Hospital. The protocol was approved by the Ethics Committee of the Institute of Geriatrics, Beijing Hospital. All subjects gave written informed consent in accordance with the Declaration of Helsinki.

## Author Contributions

RL, XZ, YY, and FL contributed conception and design of the study. SH, YC, QZ, BH, YG, and YX organized the data. SH, YC, and QZ performed the statistical analysis. SH and YY wrote the first draft of the manuscript. JW wrote sections of the manuscript. All the authors contributed to manuscript revision, read, and approved the submitted version.

## Conflict of Interest Statement

The authors declare that the research was conducted in the absence of any commercial or financial relationships that could be construed as a potential conflict of interest.
