# Supplementary material for: Anti-thrombosis Effects and Mechanisms by Xueshuantong Capsule Under Different Flow Conditions
Source: Front Pharmacol. 2019 Feb 7;10:35. doi: 10.3389/fphar.2019.00035 (PMC6374556; doi:10.3389/fphar.2019.00035)
Supplement: Supplementary file 1 [file Data_Sheet_1.docx]

**Original Figure Legends**

S1 Fig. HPLC fingerprint of the XST capsule. P1, notoginsenoside R_1_; P2, ginsenoside Rg_1_; P3, ginsenoside Re; P4, ginsenoside Rb_1_; P5, ginsenoside Rd.

S2 Fig. The introduction of Bioflux1000 flow system for cellular Interactions assays. A) Photograph of the Bioflux1000 flow system used to carry out shear stress experiments. Tubing connecting the pressure controller to the plate was fed into a time-lapse microscope on which the microfludic channels were visualized. B) The plate schematic demonstrates the setup of a 48 well, 24 channel plate within which media flows from the inlet well (I) to outlet (O) wells in parallel. C) The channel schematic drawing demonstrates Inlet wells (I) and outlet well (O) which serve as independently controlled fluid reservoirs. D). Schematic depiction of Fluxion’s Well Plate Microfluidics. Fluid reagents are loaded into wells on the plate, and controlled pressures are applied via the Interface to move them throughout the channels, which generate controlled shear stress. The channels run on the bottom of the plate from well to well.
