# Supplementary figures and images for: Anti-thrombosis Effects and Mechanisms by Xueshuantong Capsule Under Different Flow Conditions
Source: Front Pharmacol. 2019 Feb 7;10:35. doi: 10.3389/fphar.2019.00035 (PMC6374556; doi:10.3389/fphar.2019.00035)

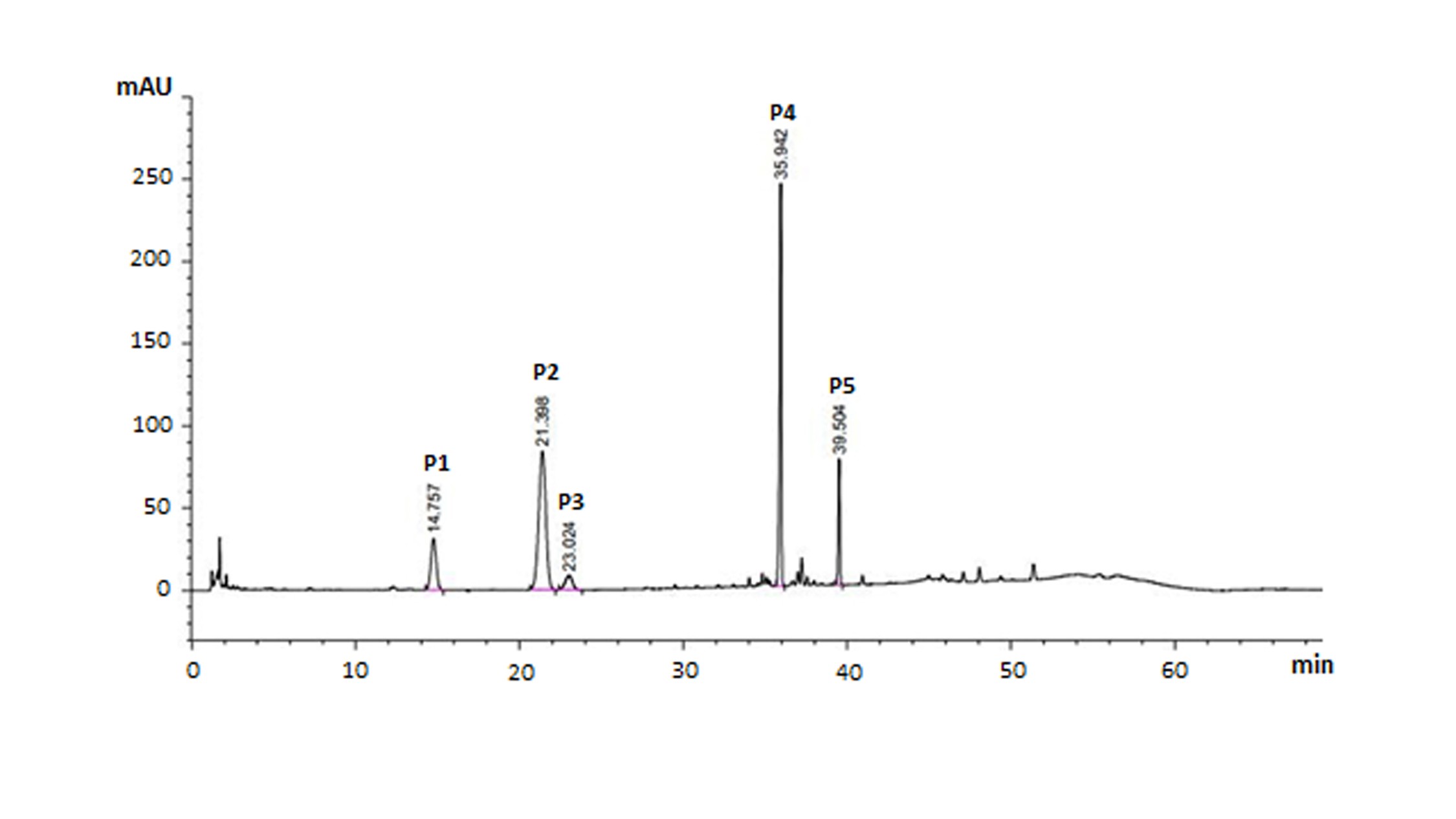

Supplement: Supplementary file 2 [file Image_1.TIF]

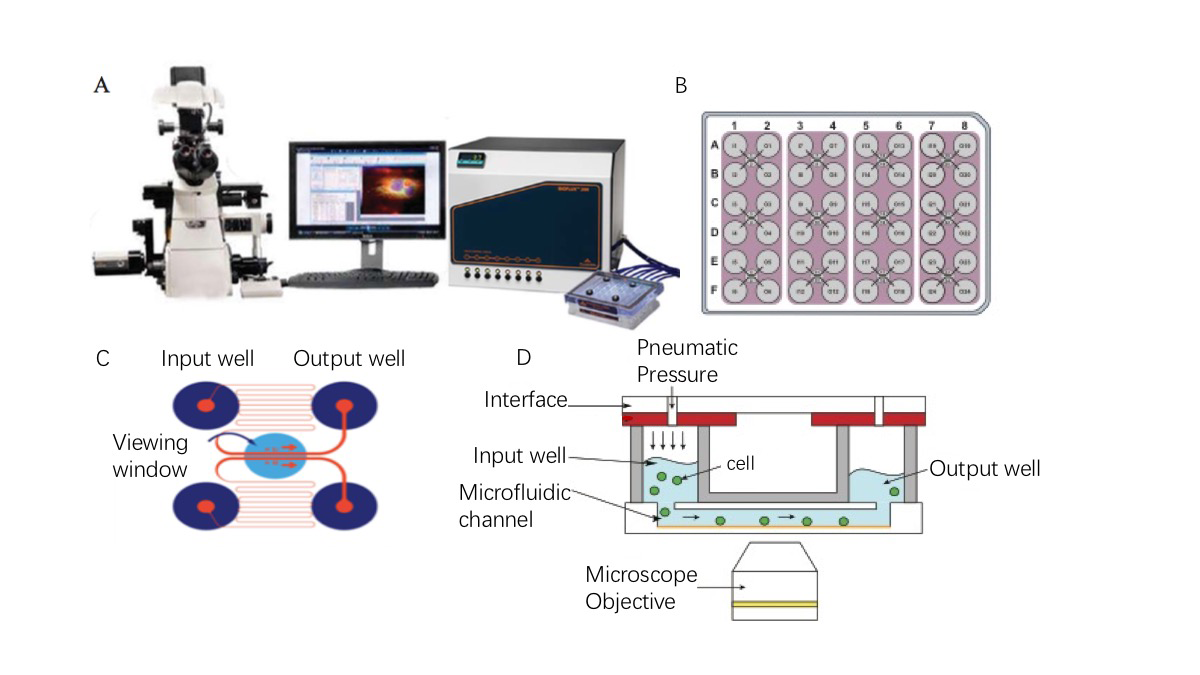

Supplement: Supplementary file 3 [file Image_2.TIF]
